# Unilateral biportal endoscopy for the treatment of symptomatic spinal epidural lipomatosis: a case report and literature review

**DOI:** 10.3389/fsurg.2025.1580499

**Published:** 2025-06-05

**Authors:** Yahao Li, Hong Guo, Zihang Li, Yucheng Wang, Zhenyu Tang, Hongwei Li, Hong Jiang, Jintao Liu, Yuxiang Dai, Guangye Zhu, Pengfei Yu

**Affiliations:** ^1^ Department of Orthopaedic Surgery, Suzhou TCM Hospital Affiliated to Nanjing University of Chinese Medicine, Suzhou, Jiangsu, China; ^2^ Nanjing University of Chinese Medicine, Nanjing, Jiangsu, China

**Keywords:** unilateral biportal endoscopy, symptomatic spinal epidural lipomatosis, minimally invasive, case report, spine

## Abstract

**Background:**

Spinal epidural lipomatosis (SEL) is rare and often ignored or misdiagnosed. Traditional open surgery is considered the standard procedure for treating symptomatic SEL. However, open surgery is often associated with substantial trauma and a long recovery period.

**Case presentation:**

A 37-year-old female patient was diagnosed with SEL and underwent unilateral biportal endoscopy (UBE) after failure of conservative treatment. The surgery was performed successfully with an estimated blood loss of 20 ml and a whole operation time of 60 min. The patient experienced significant relief of her neurologic symptoms and was discharged 2 days postoperatively. She reported no symptoms other than mild weakness of the low back at the last follow-up 12 months postoperatively. UBE may be an effective alternative to open surgical treatment for symptomatic SEL with the advantages of minimal invasion and quick recovery.

**Conclusions:**

SEL is not restricted to the commonly involved lumbosacral region and may occur in other segments of the spine, which should be considered during diagnosis. The advantages of UBE for treating SEL, including minimal invasiveness, muscle preservation, rapid recovery, clear visualization, and effective decompression, make it a viable surgical option; however, its long-term efficacy and safety require further validation.

## 1 Introduction

Spinal epidural lipomatosis (SEL) refers to abnormal accumulation of epidural fat tissue in the spinal canal, resulting in radicular symptoms in the corresponding intervertebral segment. The incidence of SEL is approximately 1.1% (1). The symptoms of SEL are similar to those of lumbar disc herniation, including low back pain and pain and numbness in the lower limbs (2). Therefore, the diagnostic rate is reported to be only 8% in clinical practice, which potentially delays the treatment of patients with SEL (3). Bed rest and nonsteroidal drugs are usually used for conservative treatment; however, surgical intervention should be considered when conservative treatment fails. Traditionally, open surgeries include laminectomy, lpectomy, discectomy, and intervertebral fusion (4). Nevertheless, disadvantages such as intervertebral instability, traumatic injury, bleeding, postoperative pain, and long recovery times have often been reported in the treatment of patients with open surgeries in recent studies (5). In recent years, with the development of minimally invasive surgical techniques, unilateral biportal endoscopy (UBE) has gradually gained popularity among spinal surgeons. Compared to traditional open surgeries, UBE has the advantages of less harm to anatomical structures, less postoperative pain, and a shorter recovery time in the treatment of lumbar disc herniation or lumbar spinal stenosis (6, 7). However, the application of the UBE technique in the treatment of SEL has rarely been reported. This report aims to introduce a case in which a patient with SEL was treated using the UBE technique and symptoms were significantly relieved.

## 2 Case presentation and surgical technique

### 2.1 Patient information

**Figure 1:**
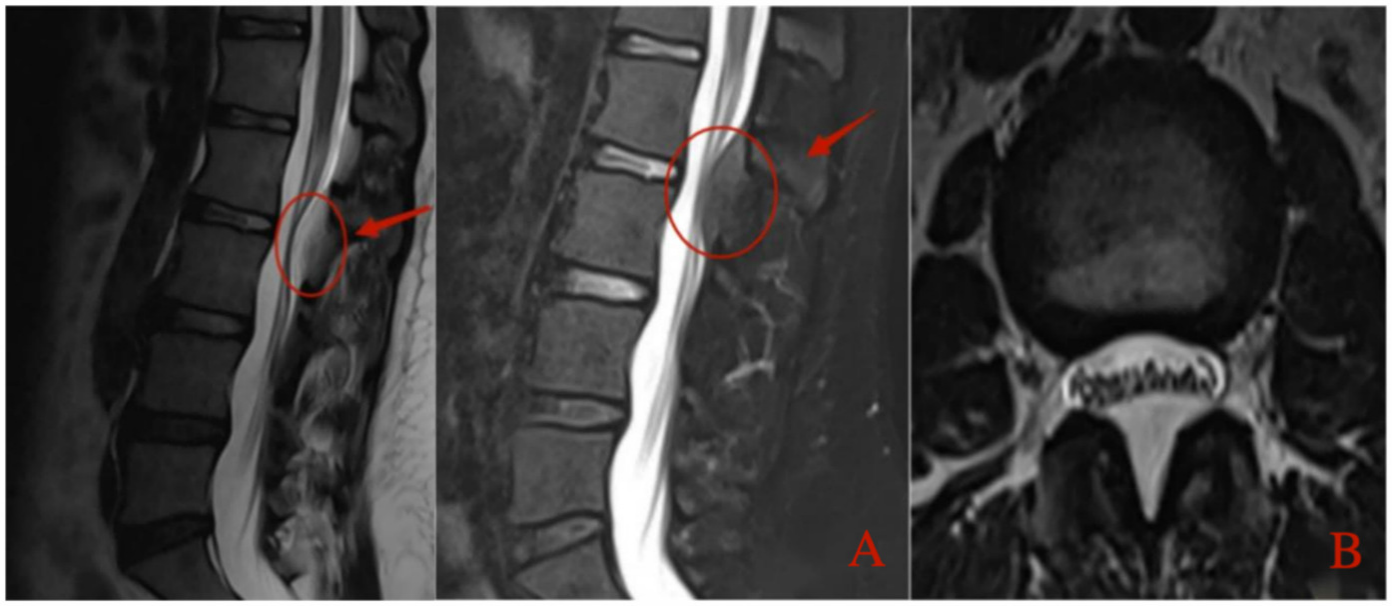
Preoperative lumbar MRI. **(A)** Sagittal T2-weighted image demonstrates a hyperintense epidural mass compressing the dorsal aspect of the dural sac at the L2–L3 level, resulting in spinal canal stenosis. **(B)** Axial T2-weighted image at the L2–L3 level shows narrowing of the spinal canal with epidural fat accumulation causing anterior displacement and compression of the dural sac.

**Figure 2:**
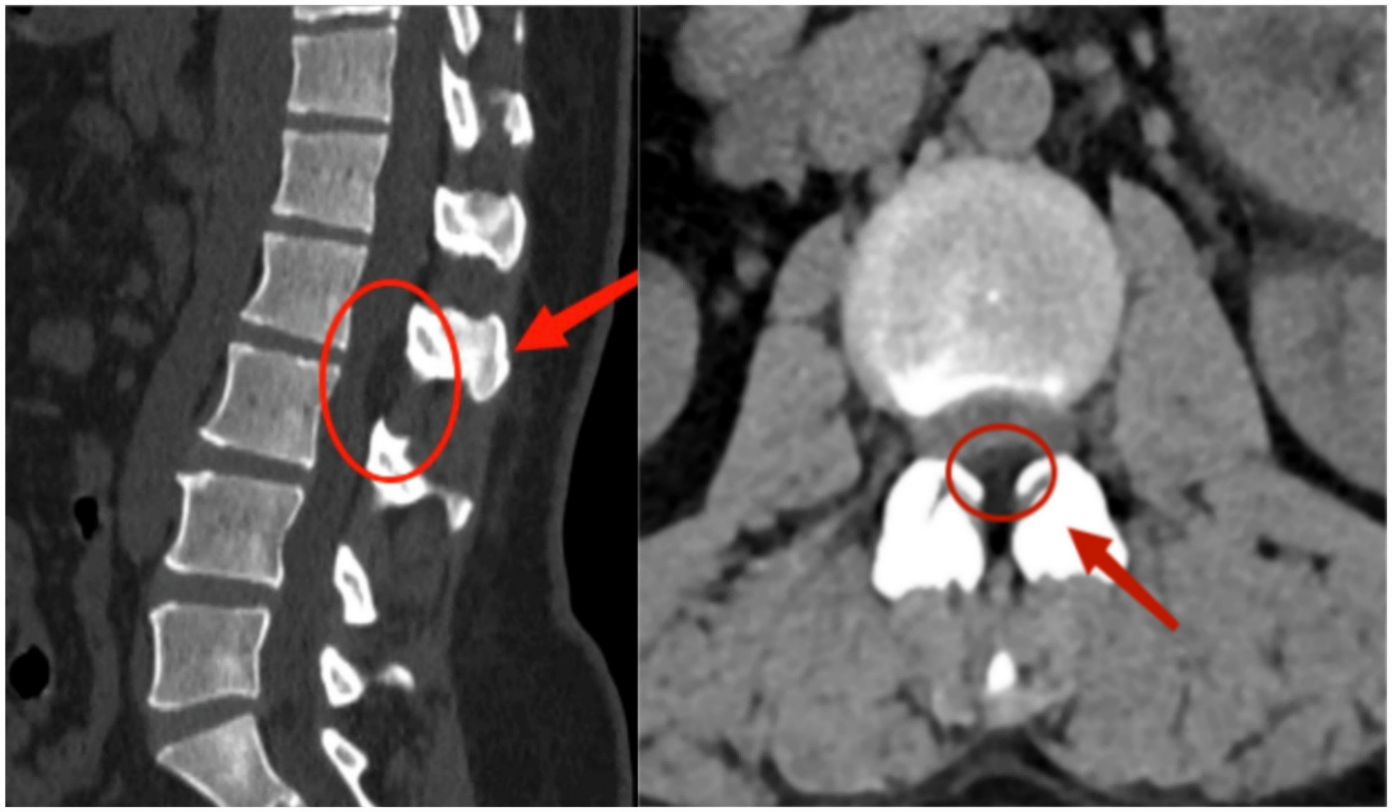
Preoperative lumbar CT imaging. Axial CT image at the L2–L3 level reveals fat-density shadows located at the dorsal part of the dural sac, resulting in spinal canal stenosis.

**Figure 3:**
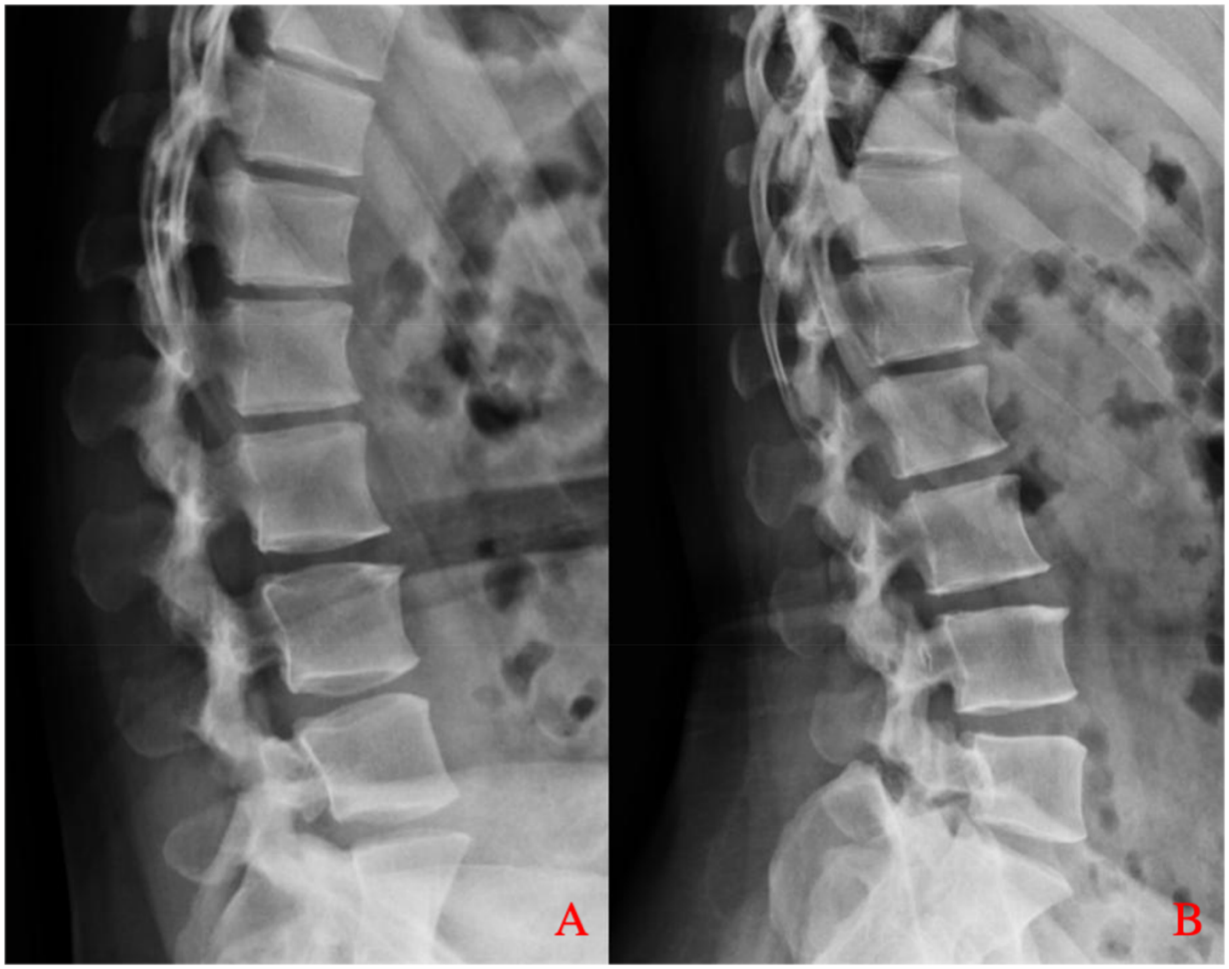
Dynamic lumbar spine radiographs. **(A)** Flexion and **(B)** extension views demonstrate preserved alignment at the L2–L3 level, with no signs of abnormal translation or angulation, indicating no significant segmental instability.

A 37-year-old female patient complained of low back pain with right lower extremity pain and numbness for 3 years. She described her pain as profoundly impairing her ability to carry out her daily tasks and expressed growing anxiety due to the persistent numbness and the fear of lasting nerve damage. She was obese with a body mass index (BMI) of 30.48 kg/m^2^. Her pain worsened when walking long distances or when she was tired after work. Physical examination revealed decreased tendon reflexes in her right knee and decreased skin sensation in front of her right thigh. After being admitted to our hospital, she underwent detailed imaging examinations and was managed conservatively using dexamethasone and mannitol. However, her symptoms were not alleviated after conservative treatment. Magnetic resonance imaging (MRI) of the lumbar spine revealed a space-occupying lesion at the dorsal side of the dural sac in the spinal canal, resulting in spinal stenosis at the L2–L3 segment (Figure 1). The lesion appeared smooth and fusiform and exhibited homogeneously hyperintense signal characteristics on T1-weighted images consistent with fat tissue. Similar findings were found on computed tomography (CT) of the lumbar spine (Figure 2). Dynamic radiographs revealed no significant instability at the L2–L3 segment (Figure 3). Based on her symptoms, physical examinations, and imaging findings, the space-occupying lesion on the dorsal side of the dural sac at the L2–L3 segment was believed to be responsible for her symptoms. After obtaining informed consent, we performed decompression and resection of the epidural space-occupying lesion using the UBE technique.

### 2.2 Surgical technique

**Figure 4:**
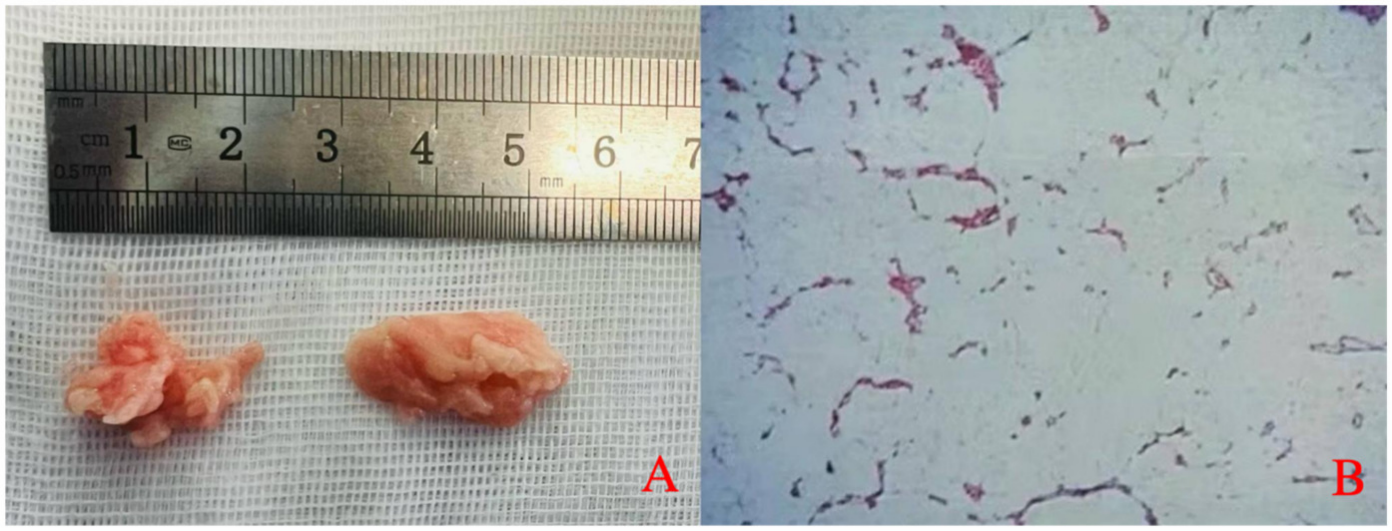
**(A)** Gross appearance of the excised epidural tissue. **(B)** Histopathological examination reveals mature adipose tissue with focal areas of calcification.

Under general anesthesia, the patient was placed in the prone position on a radiolucent operating table. The responsible segment was confirmed using C-arm fluoroscopy. On the right side of the posterior median line, two transverse incisions were made on the proximal and distal sides centered on the L2–L3 intervertebral space. The proximal incision was used as the operating portal, while the distal incision was used as the viewing portal. The skin and fascia were sequentially incised. Endoscopic instruments were placed on the surface of the laminae and the interlaminar space. The muscle tissue of the laminae and the interlaminar space was dissected, and the ligamentum flavum between the laminae was resected. The lower margin of the proximal lamina and the upper margin of the distal lamina were removed via an endoscopic high-speed drill and rongeur, thereby enlarging the interlaminar space. The epidural space was filled with fatty soft tissue, and the right nerve root and dural sac were compressed. The fatty soft tissue was carefully dissected and removed using forceps (Figure 4A). Rhythmic pulsation of the dura mater and nerve root in the endoscopic view was considered an indicator of adequate decompression. The working cannula and endoscope were removed after careful hemostasis. Finally, the skin incision was closed using absorbable sutures.

### 2.3 Postoperative imaging examinations and follow-up visit

**Figure 5:**
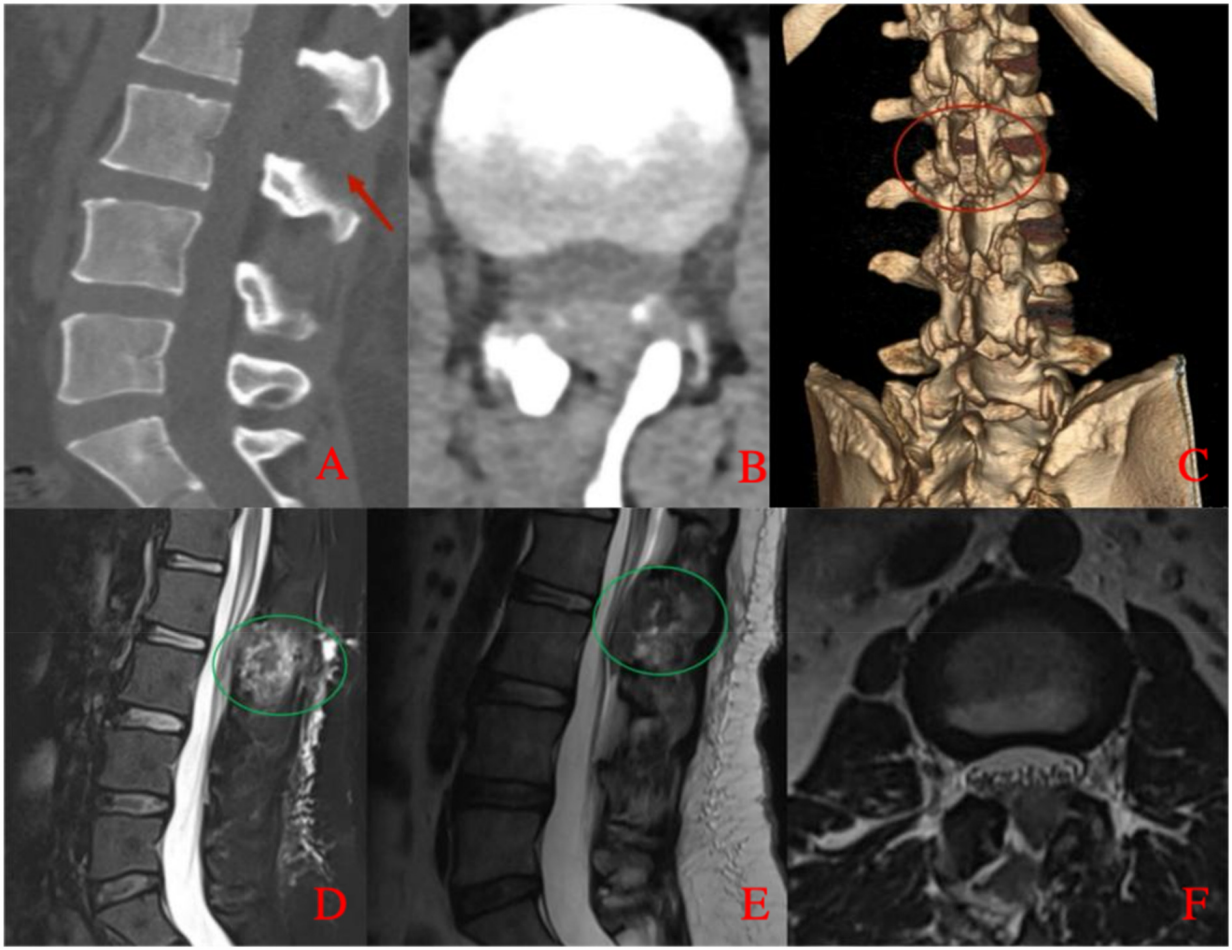
Postoperative lumbar CT and MRI. **(A)** Sagittal CT image shows no epidural mass at the L2–L3 level with evidence of partial laminectomy. **(B)** Axial CT image demonstrates adequate spinal canal decompression with no residual epidural compression. **(C)** Three-dimensional CT reconstruction illustrates the extent of laminectomy. **(D)** Sagittal fat-suppressed T2-weighted MRI confirms resolution of dorsal compression and shows postoperative changes with a small residual hematoma. **(E)** Sagittal T2-weighted MRI further demonstrates restoration of the dural sac morphology. **(F)** Axial T2-weighted MRI at the L2–L3 level shows no residual mass and adequate decompression of the spinal canal, with a small amount of postoperative hematoma.

**Figure 6:**
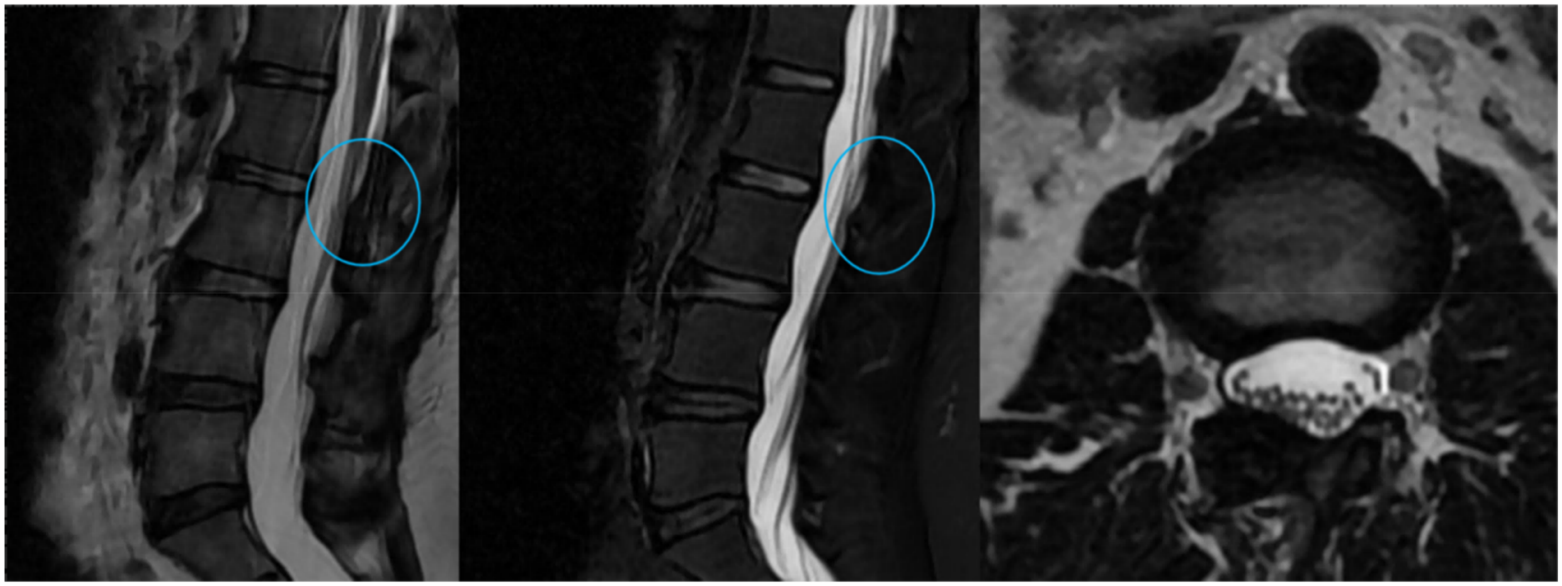
Lumbar MRI at the 12-month follow-up. Sagittal and axial T2-weighted images show no evidence of recurrence at the L2–L3 level, with sustained decompression of the dural sac.

The patient's symptoms significantly improved postoperatively. Histopathological examination of the excised tissue revealed adipose tissue with focal calcification (Figure 4B). Postoperative imaging examinations confirmed that the lesion on the dorsal side of the dural sac in the spinal canal had been removed (Figure 5). When asked about her recovery experience, the patient stated, “*The relief was immediate. By the next day, I was able to stand upright without pain, although I still experienced some lower back weakness while standing and walking.*” The patient reported no symptoms except mild weakness of the lower back, and no recurrence was found on MRI examination at the 12-month follow-up (Figure 6).

## 3 Discussion

### 3.1 Pathogenesis and diagnosis of SEL

Although the pathogenesis of SEL remains unclear, research suggests that it may be linked to several factors (8). Exogenous steroid use is the most common cause (9). Moreover, endogenous steroid-related conditions such as Cushing's syndrome and hypothyroidism have been associated with SEL (8). A recent study revealed that the BMI is positively correlated with the incidence of SEL (10), possibly because elevated inflammatory cytokines (e.g., TNF-α and IL-1β) promote adipocyte proliferation in obese patients (11). Spinal surgery can also cause epidural fat accumulation, leading to SEL (12). Some cases are idiopathic (idiopathic epidural lipomatosis), although this definition remains controversial.

The most frequent symptoms of patients with SEL included low back pain and neurogenic claudication, and these are similar to those of patients with lumbar disc herniation or spinal stenosis. Other conditions that can lead to nerve compression, such as vertebral fractures, spinal hemangiomas, abscesses, spinal tumors, disc herniation, ligamentum flavum hypertrophy, or epidural metastases, may also lead to similar clinical symptoms (13). Therefore, the diagnosis of SEL is often confusing and challenging. Imaging examinations are particularly critical. X-ray imaging is not useful for diagnosing SEL. On CT scans, SEL can manifest as a focal epidural filling defect with smooth margins and CT attenuation values between −80 and −120 HU (14). MRI is considered the most sensitive modality for the diagnosis of SEL (15). Characteristic MRI findings include the “Y-sign” or polygonal deformation of the thecal sac due to compression, which is more evident in advanced cases, whereas early detection might rely on other imaging features (16). Additionally, with high signal intensity on T1-weighted images, intermediate intensity on T2-weighted images, and fat-suppressed sequences, MRI can effectively differentiate the dura mater from accumulated fat, precisely locate the lesion, and distinguish SEL from conditions such as spinal hemangiomas and tumors.

### 3.2 Analysis of SEL localization in the case

SEL is more prevalent among male adults and most frequently occurs in the lumbosacral spine, followed by the thoracic spine (17). In this case, SEL occurred at the lumbar (L2–L3) level, which is a relatively unusual location, prompting further investigation. First, the anatomical structure of the lumbosacral region is a significant factor contributing to the predisposition for SEL. From the L1–L2 level onwards, the spinal cord gradually tapers to form the conus medullaris, with the cauda equina below. Compared to the thoracic and cervical vertebrae, the spinal canal and neural foramina of the lumbar or lumbosacral vertebrae have significantly increased space, providing room for abnormal accumulation of adipose tissue. However, despite having more anatomical space than the cervical and thoracic vertebrae, the L2–L3 level does not possess the distinct anatomical advantages of the lumbosacral region. Therefore, the occurrence of SEL may be influenced by other factors. Second, in terms of its pathophysiology, the patient in this case had a BMI of 30.48 kg/m^2^, indicating that she was obese. Obesity is associated with excess visceral fat, which can be passively stored in organs with lower fat content, such as the liver, skeletal muscles, and even the lumbar epidural space, leading to ectopic fat deposition (18). This may, to some extent, influence the location of SEL. As abdominal fat accumulation increases intra-abdominal pressure, fat cells may passively shift to surrounding areas, including the relatively higher lumbar spinal canal, resulting in SEL at the L2–L3 segment.

The occurrence of SEL at the relatively unusual L2–L3 segment may be related to factors such as the anatomical structure of the lumbar spinal canal, fat distribution, and transfer mechanisms in obese patients. Therefore, while SEL is most commonly seen in the lumbosacral region, there are reports of cases occurring in higher spinal levels. Papastefan et al. described a 9-year-old patient with multilevel (C2–C7) SEL who underwent partial laminectomy and experienced significant relief (19). However, although such cases are extremely rare, they highlight the diversity in the location of SEL and emphasize the need to consider individual patient factors and the pathophysiological mechanisms of the lesion, rather than focusing only on the common lumbosacral region.

### 3.3 Progress of treatment for SEL

For surgery-intolerant patients and those with mild symptoms, conservative management is preferred. Weight loss is the primary recommendation for all patients with SEL (20). However, if conservative treatment fails, surgery may be considered. Valcarenghi et al. (21) reported the case of a 48-year-old obese patient (BMI 37.4 kg/m^2^) with SEL who underwent sleeve gastrectomy and experienced significant symptom improvement 6 months postoperatively, underscoring the role of obesity management. Han et al. (22) also described a 53-year-old male patient with SEL underwent decompression, bone grafting, and fixation and achieved complete relief 2 years postoperatively. Laminectomy is suitable for the management of severe neurological deficits, removing excess fat, and relieving nerve compression (23). Yang et al. (5) performed laminectomy with fat removal, achieving a balance between nerve decompression and spinal canal integrity.

Minimally invasive techniques, such as endoscopic liposuction and minimally invasive spinal endoscopic surgery, have gained attention in recent years (8). Frank (24) first reported using minimally invasive liposuction to treat SEL of the L4–S1 segment. Kang et al. from South Korea (7) performed percutaneous biportal endoscopic surgery (PBES) on three patients with SEL. Using a unilateral approach, they removed part of the lamina and ligamentum flavum and then precisely excised the epidural fat tissue. Postoperative follow-up revealed significant symptom improvement in the patient, confirming the efficacy and safety of this minimally invasive endoscopic surgery for SEL. To the best of our knowledge, this is one of the few reports on the treatment of SEL using UBE. However, owing to the low prevalence of SEL and the ongoing controversy surrounding its optimal treatment, reporting relevant studies conducted in hospitals across different regions is currently important. Our study's findings provide additional clinical evidence for the treatment of SEL using UBE.

### 3.4 Advantages, technical considerations, and drawbacks of UBE treatment for SEL

In recent years, UBE has emerged as a promising technique for managing degenerative spine diseases (25). Small incisions minimize iatrogenic trauma, reduce postoperative pain, and accelerate recovery (26–28). The clear endoscopic field allows precise localization and dissection of SEL lesions while protecting neural structures (29). Furthermore, since SEL typically occurs dorsally, the posterior approach of UBE is relatively straightforward. Importantly, its gentle learning curve enables more surgeons to master the technique (25).

The critical technical considerations of UBE treatment for SEL are summarized below. First, comprehensive preoperative evaluation is essential for achieving surgical success. MRI and CT should be performed preoperatively to determine the responsible segment. Segmental stability should also be assessed using dynamic imaging. Second, establishing a precise unilateral biportal trajectory and ensuring proper visualization are critical. The surgical approach should be aligned with the location of the lesion, with either an interlaminar or foraminal entry route selected to facilitate optimal access to the affected segment. Third, during the procedure, surgeons must remove the ligamentum flavum and epidural fat to decompress the spinal canal and relieve nerve compression. Fine instruments should be used to excise fat tissue, meticulously avoiding damage to the dural sac or nerve roots. Fourth, controlling bleeding using radiofrequency ablation or hemostatic agents is crucial for ensuring a clear surgical field. Furthermore, intraoperative blood pressure fluctuations can also affect bleeding. Therefore, the surgeon needs to communicate with the anesthesiologist to achieve and maintain an optimum blood pressure within a relatively low and stable range.

Despite its advantages in clinical practice, UBE presents several challenges. Maintaining clear visualization in the surgical field is often compromised by restricted anatomical spaces and intraoperative bleeding. Surgeons must skillfully regulate irrigation pressure and suction to effectively address these challenges. The proximity of fat tissue to neural structures, particularly in cases of calcified SEL, increases the risk of iatrogenic injury and necessitates precise instrumentation. Coordinating between endoscopic visualization and instrument manipulation is crucial for preventing instrument-channel conflicts. Additionally, the small and relatively delicate tools employed in UBE are limited in efficacy for treating hard bony structures or ligaments. Furthermore, the applicability of UBE in cases involving extensive lesions involving multiple spinal levels remains uncertain due to technical constraints and a lack of extensive evidence. Potential complications, including dural tears, nerve injuries, or postoperative hematomas, require immediate management to mitigate associated risks. Postoperative care should focus on controlling inflammation via the use of anti-inflammatory agents and physical therapy, as well as preventing adhesions to optimize recovery.

## 4 Conclusions

SEL is not confined to the commonly affected lumbosacral region; thus, accurate diagnosis requires a thorough evaluation that integrates both clinical symptoms and imaging findings to avoid misdiagnosis or missed diagnosis. UBE offers significant advantages in clinical practice for the treatment of SEL, including minimal invasiveness, muscle preservation, early recovery, high-definition visualization, and effective dorsal decompression. This technique represents a promising and effective alternative for the management of SEL, particularly in patients who prioritize rapid functional recovery and minimal disruption to daily life. However, further studies with long-term follow-up are needed to validate its efficacy and safety.

## Data Availability

The original contributions presented in the study are included in the article/Supplementary Material, further inquiries can be directed to the corresponding author.
